# Synthetic mesh repair of an anterior perineal hernia following robotic radical urethrocystectomy

**DOI:** 10.1590/S1677-5538.IBJU.2016.0534

**Published:** 2017

**Authors:** Horacio J. Alvarez Garzón, Thomas Maubon, Camille Jauffret, Pierre Vieille, Brigitte Fatton, Renaud de Tayrac

**Affiliations:** 1 Department of Urology, Hospital Privado Centro Medico de Córdoba, Argentina; 2 Department of Obstetrics and Gynaecology, Nîmes University Hospital, Nîmes, France; 3 Department of Urology and Surgical Oncology, Institut Paoli Calmettes, Marseille, France

**Keywords:** Perineum, Pelvis, Cystectomy

## Abstract

**Introduction:**

Perineal hernia is a protrusion of intra-abdominal viscera through a defect in the pelvic floor and is a rare but challenging complication after extensive abdominoperineal surgery. There have been small series published after colorectal exenteration, but no cases have been reported after radical cystectomy and urethrectomy.

**Case Presentation:**

A 68 years old woman developed an anterior perineal hernia, with no vaginal prolapse, after an anterior exenteration for bladder cancer. A perineal approach with the use of a synthetic polypropylene mesh was chosen to resolve the condition. After 6 months of follow-up, the patient has no symptoms or recurrence of the anterior perineal hernia.

**Conclusion:**

To our knowledge, this case is the first report of perineal hernia after radical urethrocystectomy. Although being a case report, this article describes a potential and challenging complication after extensive anterior pelvic surgery, that could increase its incidence in the future. Literature review shows that whether perineal, abdominal or combined approach is chosen, surgery must respect hernia repair principles.

## INTRODUCTION

Perineal hernia is a protrusion of intra-abdominal viscera through a defect in the pelvic floor. Symptomatic perineal herniation is a rare but challenging complication after extensive abdominoperineal surgery. A Mayo Clinic publication, reported a prevalence of 0.34% (8 of 2732) in patients operated on between 1990 and 2000 with a minimum 5-year follow-up (1). Risk factors for perineal hernia may include wound infection, coccygectomy, previous hysterectomy, pelvic irradiation, redundant small bowel mesentery, female pelvis, excision of or failure to reapproximate the elevators, perineal wounds left open postoperatively, placement of drains through the perineal wound itself instead of through separate stab incisions and tobacco use (2, 3).

To our knowledge, no cases have been published following radical cystectomy and urethrectomy. This article reports a perineal hernia after this surgery and its surgical management using a synthetic mesh through a perineal approach.

## CASE PRESENTATION

A 68 years old female, with a history of smoking habit, was diagnosed with high-grade muscle invasive urothelial bladder carcinoma after undergoing transurethral bladder resection for gross hematuria. The tumor involved the left lateral wall and the bladder neck. A positron emission tomography showed enlarged iliac lymph nodes and a neoadjuvant chemotherapy based on platinum (M-VAC: methotrexate, vinblastine, doxorubicin and cisplatin) was prescribed. No pelvic irradiation was necessary.

A robotic radical cystectomy, hysterectomy with bilateral adnexectomy, extended pelvic lymphadenectomy and urethrectomy with heterotopic Bricker urinary diversion was performed. Due to the locally advanced disease prior to chemotherapy a broad resection was performed including a complete urethrectomy.

The postoperative recovery was marked by a delayed vestibule healing and an inguinal hematoma that required surgical drainage. The pathologic examination retrieved a high-grade muscle-invasive bladder urothelial carcinoma, and a final TNM score of pT2 No M0 with negative surgical margins.

After one month of the surgery, an anterior perineal bulge appeared during efforts, increasing its volume during the following months. The patient had severe discomfort, despite not complaining of pain or digestive symptoms.

**Figures 1:**
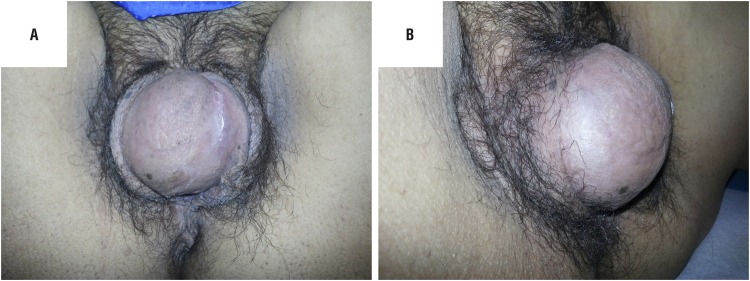
A and B) Preoperative perineal hernia frontal and lateral appearance during Valsalva effort.

**Figure 2:**
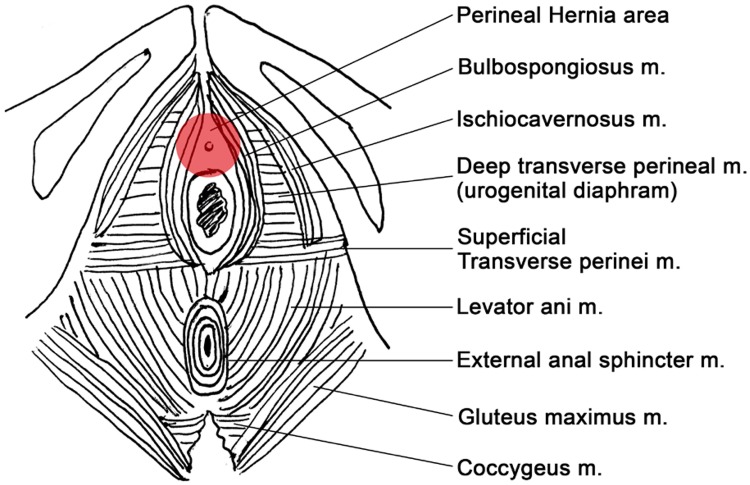
Anatomy of perineum and location of this anterior perineal hernia.

Upon physical examination, an anterior perineal hernia was observed between the ischiopubic rami, at the level of the vestibulum with labia majora bulging but without vaginal prolapse (Figures 1 A and B and Figure-2). The content of the hernia was reducible and the skin covering the hernia had a diminished thickness.

**Figure 3:**
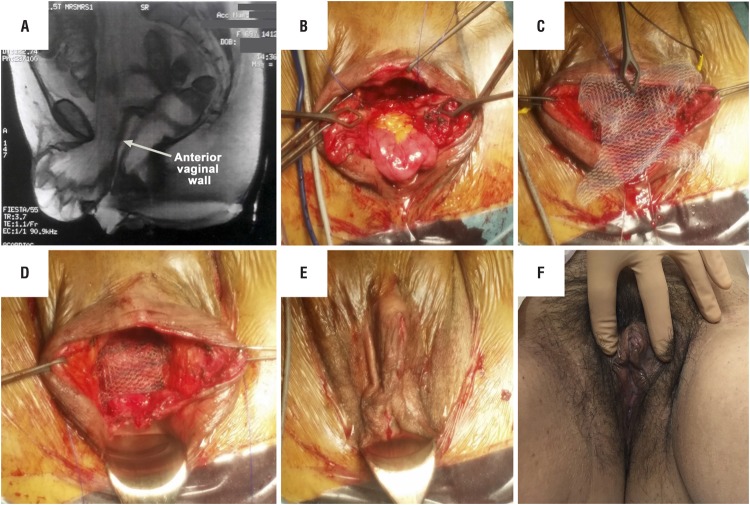
A) Sagital Magnetic resonance image. B) Intraoperative view of the opened sac of anterior enterocele. C) Four- arms polypropylene mesh. D) Intraoperative view of the fixed mesh. E) Intraoperative final result. F) Postoperative exam with Valsava effort after 6 months of surgery.

A magnetic resonance scan showed a small bowel content at the level of the perineal hernia, without vaginal prolapse (Figure-3A). No radiologic signs of cancer recurrence were observed.

A multidisciplinary meeting was held and, based on the patient's discomfort, possible complications, and after patient's consent, surgery was scheduled.

Under general anesthesia, with the patient placed in a lithotomy position with Allen stirrups, broad-spectrum antibiotic provided, an infiltration of the perineal skin with a solution of lidocaine and 1% adrenaline was performed. Sub - clitoridian vestibular and lower portion of the anterior vaginal wall incision. Dissection of the voluminous enterocele and opening of the peritoneum sac (Figure-3B).

Dissection was extended behind the fat tissue of the labia majora and to the level of the ilio-pubic rami, where two polypropylene 2/0 sutures were placed bilaterally on the inferior pubic ligaments. The peritoneum was closed with a purse-string polydioxanone 1 suture, and reinforcement of 2/0 poliglecaprone interrupted sutures. The urogenital diaphragm and bulbo-spongiosus muscles were then sutured in the midline using 2/0 poliglecaprone interrupted sutures to cover the peritoneum sac.

A transobturator light weight polypropylene mesh (Surgimesh^®^, Aspide Medical Laboratoire, La Talaudière, France) was placed, fixed to the iliopubic rami with the 2/0 polypropylene sutures and with one arm placed through the transobturator foramen on each side (Figures 3C and D). Finally, the mesh was attached to the anterior vaginal wall with polypropylene 2/0 interrupted sutures. This type of mesh and fixation was chosen since the vaginal vault was not accessible through this surgical approach. The mesh was partially covered medially by labia majora fat tissue using absorbable sutures. Finally, the skin and vestibular mucosa were closed with a 3/0 poliglecaprone running suture (Figure-3E).

After a follow-up of 6 months, there was no evidence of recurrent hernia and no scar defect (Figure-3F).

## DISCUSSION

Perineal hernia is an infrequent but challenging event after extensive abdominoperineal surgery, mainly in the posterior compartment. The publications on this subject are scarce and usually based on single or small case series. Incidence ranges between 0.6% and 7% (4–6). To our knowledge, there are no publications of anterior perineal hernia after radical cystectomy and urethrectomy.

Usual presentation is that of a soft, reducible mass, along with discomfort and bulging in the perineum, which was the case of our patient. Complications may include skin breakdown, bowel obstruction due to incarcerated small intestine, entero-cutaneous fistula or the extreme presentation of hernia rupture with small-bowel prolapse and/or evisceration (7).

As previously stated, risk factors for perineal hernia (1) may include wound infection, previous hysterectomy, pelvic irradiation, redundant small bowel mesentery, female pelvis, failure to reapproximate the elevators, perineal wounds left open postoperatively, placement of drains through the perineal wound itself instead of through separate stab incisions, and tobacco use (2, 3). In our case, the extensive resection in order to accomplish negative margins on the urethrectomy might have been the cause. Other reason could have been the lacking of force feed-back sensation in robotic approach.

Although diagnosis might be evident, the use of imaging studies, as computed tomography or magnetic resonance imaging may provide information on hernia content, soft tissue surroundings and exclude recurrent malignancy before deciding a reconstructive surgery.

Based in digestive surgery literature (2, 3, 8), a variety of methods for repair have been described, most as isolated case reports or small case series because of the relative infrequency of the condition.

The basic principles of hernia repair must be followed: exposure and mobilization of the hernia sac, reduction of its content, excision of the sac, and repair of the defect. There is no accepted consensus as to the preferred approach, perineal, abdominal or combined.

Regardless of chosen surgical approach, the main goal is to close the hernia defect, that can be achieved by reapproximation of the tissues with non-absorbable suture or in poor quality autologous tissues, by using a mesh support (9–11). When large anatomic defects are present or when the use of synthetic mesh implant is contraindicated, autologous fascia lata flaps or myocutaneous rotational flaps can be used (12). Whether the ideal material is an autologous graft or flap, synthetic mesh, or a bioprosthetic mesh has not been well established.

In our case, due to the urinary diversion, the perineal approach was chosen. Furthermore, since the patient had no previous radiotherapy, a polypropylene mesh was used in order to ensure a correct and permanent support of the pelvic floor defect, thus diminishing the risk of hernia recurrence.

Although being a rare condition, anterior perineal hernia should be suspected in patients who complain of perineal bulge after extensive abdominoperineal surgery. To our knowledge, this is the first case reported of anterior perineal hernia after radical cystectomy and urethrectomy.

## INFORMED CONSENT

Informed consent was obtained from the patient for publication of this article and accompanying figures.
